# Prognostic Effect of Primary Recurrence Patterns in Squamous Cervical Carcinoma After Radical Surgery

**DOI:** 10.3389/fonc.2022.782030

**Published:** 2022-04-11

**Authors:** Zongkai Zhang, Long Jiang, Rui Bi, Xiaohua Wu, Guihao Ke, Jun Zhu

**Affiliations:** ^1^ Department of Oncology, Shanghai Medical College, Fudan University, Shanghai, China; ^2^ Department of Anesthesia, Zhongshan Hospital Fudan University Shanghai Cancer Center, Shanghai, China; ^3^ Department of Pathology, Fudan University Shanghai Cancer Center, Shanghai, China; ^4^ Department of Gynecologic Oncology, Fudan University Shanghai Cancer Center, Shanghai, China

**Keywords:** cervical cancer, recurrence patterns, therapy for relapse, locoregional recurrence, distant metastasis

## Abstract

**Objective:**

To examine the effect of primary recurrence patterns on the prognosis of squamous cervical cancer after initial treatment.

**Methods:**

Primary recurrence patterns and prognostic factors were examined in stage IB-IIA cervical cancer patients after initial treatment. Recurrence site (locoregional recurrence and distant metastasis or in-field and out-field recurrence for patients receiving adjuvant radiotherapy) and subtype (nodal and organ recurrence) were examined. Clinicopathological characteristics and survival rates were evaluated to generate a prognostic nomogram.

**Results:**

A total of 472 patients were included. The median follow-up period, 5-year overall (OS) rate, and median OS were 59.1 months, 33.7%, and 24.0 months, respectively. Overall, 38.8% and 61.2% of the patients had locoregional recurrence and distant metastasis, respectively, and survival rates were comparable in these groups. Patients with nodal recurrence had better OS than those with organ recurrence (38.3% vs 30.7%, respectively; P = 0.001). Patients not receiving adjuvant radiotherapy had increased risk of pelvic recurrence [odds ratio (OR) = 0.148; 95% confidence interval[(CI): 0.075–0.291, *P* = 0.000]. Positive lymph-vascular space invasion (OR= 1.928; 95% CI: 1.151–3.229, *P* = 0.013) and no chemotherapy (OR = 0.521; 95% CI: 0.317–0.733, *P* = 0.040) increased the risk of distant metastasis. Positive lymph node status after initial treatment were associated with nodal recurrence (OR = 3.729; 95% CI: 1.838–7.563, *P* = 0.000), while elevated preoperative squamous cell carcinoma antigen (SCC-Ag) levels were associated with organ recurrence (OR = 1.642; 95% CI: 1.325–2.265, *P* = 0.002). Recurrence subtype, therapy for relapse, the International Federation of Gynecology and Obstetrics stage, adjuvant radiotherapy, preoperative SCC-Ag levels, and risk subgroup were independently associated with OS.

**Conclusions:**

Primary recurrence patterns were associated with specific clinicopathological characteristics of cervical cancer. Recurrent cervical cancer prognosis was mainly affected by recurrence location and subtype.

## Introduction

Cervical cancer is the fourth most common gynecologic cancer worldwide and a leading cause of cancer-related deaths in developing countries (1). The incidence of cervical cancer has decreased owing to extensive screening and vaccination programs, with the latter targeting patients with high-risk human papilloma virus genotypes (2, 3). Standard treatment for early-stage cervical cancer [International Federation of Gynecology and Obstetrics (FIGO 2009) stage IB-IIA] includes radical surgery with or without adjuvant radiotherapy (RT) (4, 5). Consecutive editions of clinical guidelines have improved cervical cancer management, thus increasing remission rates and decreasing the risk of relapse; these changes have resulted in recently reported 5-year overall survival (OS) rates of early stage cervical cancer in the range of 70%–90% (6, 7). This progress notwithstanding, approximately 10%–15% of patients with early-stage disease experience recurrence (8).

Cervical cancer recurrence patterns may vary; however, they generally include locoregional recurrence (LR) and distant metastasis (DM). Recurrent disease management requires a personalized approach depending on the site of recurrence, which may determine prognosis. Previous studies have shown that patient prognosis after initial treatment was associated with clinicopathological characteristics, recurrence patterns, and relapse therapy type (9–11). However, few studies have examined risk factors for recurrence and the relationship between different recurrence patterns and prognosis. This study aimed to examine patients experiencing cervical cancer recurrence to identify their clinicopathological characteristics, recurrence patterns, and treatment type received, and to evaluate the relationship between different clinicopathological characteristics and recurrence patterns; this study aimed to examine the impact of recurrence patterns on prognosis.

## Materials and Methods

We retrospectively extracted data of 472 patients with recurrent cervical cancer who underwent standard abdominal radical hysterectomy and pelvic lymph node (LN) dissection at the Department of Gynecologic Oncology, Fudan University Shanghai Cancer Center, China, between May 2006 and January 2014. All included patients had histologically confirmed squamous cell carcinoma and the 2009 FIGO stage IB–IIA disease after initial treatment. All patients provided consent for their data to be used for research purposes. Data on clinicopathological and prognostic characteristics, including age at diagnosis, the FIGO stage, postoperative pathological findings[histological type, tumor grade, lymph-vascular space invasion (LVSI) status, tumor volume, and LN status], treatment modalities, date and type of recurrence, and date of death or last follow-up, were collected. The study was approved by the Ethics Committee of Fudan University Shanghai Cancer Center, Shanghai, China.

### Adjuvant Therapy

Patients with intermediate-risk factors that met the Sedlis criteria, including tumor diameter, depth of stromal invasion (DSI), or LVSI status, and patients with more than one high-risk factor (including parametrial involvement, positive LN, or positive surgical margins) received adjuvant RT (ART) or concurrent chemoradiotherapy. Patients received pelvic intensity-modulated radiotherapy with computed tomography (CT) planned. Target delineation was based on the Radiation Therapy Oncology Group Consensus Guidelines (12, 13). Concurrent cisplatin was administered weekly at a dose of 40 mg/m^2^. Brachytherapy was administered among patients with vaginal margin invasion. Patients with one or more high-risk factors received 4–6 cycles of paclitaxel (135 mg/m^2^) and carboplatin (AUC = 5) on day 1.

### Recurrence Classification and Treatment

Recurrence was confirmed based on findings from biopsy and/or imaging-based examinations such as CT, magnetic resonance imaging (MRI), or positron emission tomography (PET) scanning, obtained ahead of treatment planning. LR was defined as isolated pelvic recurrence, including vaginal recurrence with or without pelvic LN recurrence. DM was defined as distant site failure at either an organ or LN site. Multiple-site recurrence was defined as recurrence both inside and outside the pelvis; this type of recurrence was classified as DM. In-field and out-field recurrences were defined as recurrence inside or outside the pelvis for patients after ART. LN recurrence (LNR) included bilateral upper neck, supraclavicular, mediastinal, celiac, and pelvic and inguinal regions. Organ recurrence included recurrences in the vagina and other organs. Therapy for relapse included external beam radiation therapy and brachytherapy, surgery, and systemic chemotherapy. RT and surgery were classified as local therapy.

### Statistical Analyses

Patients’ demographic and clinicopathological characteristics and treatments for recurrent disease were reported as frequencies. Univariable survival curves were created using the Kaplan-Meier method. Between-group comparisons were performed using the log-rank test. Cox proportional hazards regression was used for univariate and multivariate analyses. Variables with p-values of <0.10 in univariate analysis were included in multivariate analyses. For all statistical tests, two-tailed p-values of <0.05 were considered significant. A prognostic nomogram was generated based on multivariable analysis results. Harrel’s concordance index (C-index) and a calibration curve were used to evaluate nomogram performance. The accuracy and reliability of the recurrence model were evaluated based on time-dependent receiver operating characteristic curves. All statistical analyses were performed in SPSS version 25.0 (SPSS, Chicago, IL) and R software ver. 4.0.5.

## Results

This study included 472 women who underwent pelvic LN dissection for cervical squamous cell carcinoma with FIGO stages IB-IIA. The patients’ median age was 47 (range: 22–77) years. All tumors were pathologically staged after radical surgery and included stages IB1 (n = 112, 23.7%), IB2 (n = 61, 12.9%), IIA1 (n = 162, 34.3%), and IIA2 (n = 137, 29.0%). Preoperative squamous cell carcinoma antigen (SCCA) levels of ≥2.55 ng/mL, bulky tumor size of ≥4 cm, DSI greater than 1/2 thickness, positive LVSI, and parametrial invasion were observed in 62.0% (n = 240), 56.8% (n = 268), 85.8% (n = 400), 63.0% (n = 289), and 14.4% (n = 64) of the patients, respectively. Thirty-eight (8.5%) patients had positive vaginal margins. Positive LN (PLN) was detected in 224 (50.3%) patients. The median number of harvested LNs was 23 (range: 7–77); a single (range: 0–37) PLN was harvested. A total of 356 (75.4%) patients underwent ART. Among 305 (85.0%) patients who underwent chemotherapy, 204 (64.2%) received concurrent chemotherapy (**Table 1**).

**Table 1:** Baseline characteristic.

	N (%)
**Age**	
<60	422 (89.4%)
≥60	50 (10.6%)
**Menopause**	
Yes	173 (36.7%)
No	299 (63.3%)
**FIGO Stage**	
IB1	112 (23.7%)
IB2	61 (12.9%)
IIA1	162 (34.3%)
IIA2	137 (29.0%)
**ECOG**	
0~1	430 (91.1%)
2~3	42 (8.9%)
**SCCA**	
<2.55	147 (38.0%)
≥2.55	240 (62.0%)
**Tumor size**	
<4cm	204 (43.2%)
≥4cm	268 (56.8%)
**Invasion depth**	
<1/2	66 (14.2%)
≥1/2	400 (85.8%)
**LVSI**	
Positive	289 (63.0%)
Negative	170 (37.0%)
**Parametrium**	
Positive	64 (14.4%)
Negative	379 (85.6%)
**Surgery margin**	
Positive	38 (8.5%)
Negative	409 (91.5%)
**Risk**	
Intermediate	188 (41.8%)
High	262 (58.2%)
**Adjuvant radiotherapy**	
Yes	356 (75.4%)
No	116 (24.6%)
**Chemotherapy**	
Yes	305 (85.0%)
No	54 (15.0%)
**Positive lymph nodes**	
Yes	224 (50.3%)
No	221 (49.7%)
**Recurrence location**	
LR	183 (38.8%)
DM	289 (61.2%)
Pelvic with distant recurrence	45 (9.5%)
**Recurrent type**	
Lymphatic	123 (26.1%)
Organic	349 (73.9%)
**Lymphatic recurrence site**	
Supraclavicular nodes	39 (8.3%)
Mediastinal nodes	28 (5.9%)
Para aortic nodes	24 (5.1%)
Retroperitoneal nodes	35 (7.4%)
Inguinal nodes	20 (4.2%)
**Organic recurrence site**	
Vaginal	264 (55.9%)
Liver	25 (5.3%)
Lung	119 (25.3%)
Bone	79 (16.7%)
**Treatment for lymph nodes**	
Radiotherapy	54 (44.0%)
Chemotherapy	51 (41.5%)
Surgery	17 (13.8%)
**Treatment for metastatic organ**	
Radiotherapy	93 (26.6%)
Chemotherapy	224 (64.2%)
Surgery	32 (9.2%)

ECOG, The Eastern Cooperative Oncology Group; SCCA, serum squamous cell carcinoma antigen; LVSI, lymph-vascular space invasion; LR, locoregional recurrence; DM, distant metastasis.

### Recurrence Patterns and Prognostic Factors

LR and DM accounted for 38.8% (183/472) and 61.2% (289/472) of recurrence cases, respectively. A total of 26.1% (n = 123) and 73.9% (n = 349) of failures were LNR and organ recurrence, respectively (**Table 2**).

**Table 2:** Univariate logistic regression models for different recurrence sites and subtypes.

Subgroup	Reference	LR		DM		LNR		Organ recurrence	
		OR (95CI)	P	OR (95CI)	P	OR (95CI)	P	OR (95CI)	P
**Age**									
>=60	<60	1.291 (0.717~2.324)	0.395	0.860 (0.475~1.560)	0.620	0.508 (0.232~1.116)	0.092	1.967 (0.896~4.315)	0.092
**FIGO stage**									
IIA	IB	0.949 (0.653~1.380)	0.784	1.207 (0.823~1.770)	0.334	1.004 (0.655~1.538)	0.986	1.004 (0.655~1.538)	0.986
**Tumor size**									
>=4cm	<4cm	1.488 (1.032~2.145)	0.033	1.381 (0.951~2.007)	0.090	1.593 (1.040~2.440)	0.032	1.593 (1.040~2.440)	0.032
**Lymph nodes**									
N+	N−	1.939 (1.330~2.826)	0.001	2.346 (1.586~3.473)	<0.001	4.630 (2.871~7.466)	<0.001	4.630 (2.871~7.466)	<0.001
**SCCA**									
>=2.55	<2.55	1.704 (1.466~2.063)	0.010	1.555 (1.023~2.365)	0.039	2.440 (1.469~4.052)	0.001	2.440 (1.469~4.052)	0.001
**DSI**									
>=1/2	<1/2	2.096 (1.223~3.593)	0.007	2.857 (1.674~4.877)	<0.001	1.944 (0.982~3.850)	0.056	1.944 (0.982~3.850)	0.056
**LVSI**									
Positive	Negative	2.506 (1.697~3.700)	<0.001	2.373 (1.606~3.506)	<0.001	4.984 (2.859~8.689)	<0.001	4.975 (2.857~8.696)	<0.001
**Parametrial**									
Positive	Negative	2.102 (1.202~3.678)	0.009	2.236 (1.210~4.130)	0.010	2.462 (1.424~4.255)	0.001	2.462 (1.424~4.255)	0.001
**Surgical margin**									
Positive	Negative	0.668 (0.339~1.318)	0.245	0.965 (0.489~1.906)	0.919	1.150 (0.551~2.398)	0.710	1.150 (0.551~2.398)	0.710
**ART**									
Yes	No	0.161 (0.098~0.265)	<0.001	0.733 (0.403~1.332)	0.308	0.282 (0.152~0.524)	<0.001	0.518 (0.250~1.074)	0.077
**Chemotherapy**									
Yes	No	0.645 (0.361~1.152)	0.139	0.193 (0.123~0.304)	<0.001	1.930 (0.931~4.000)	0.077	0.282 (0.152~0.524)	<0.001

SCCA, squamous cell carcinoma antigen; LVSI, lymph-vascular space invasion; DSI, deep stromal invasion; ART, adjuvant radiotherapy.

RT, systemic chemotherapy, and surgery for relapse accounted for 44.0% (54/123), 41.5% (51/123), and 13.8% (17/123) of therapies for LNR, respectively. The corresponding rates for organ recurrence were 26.6% (93/349), 64.2% (224/349), and 9.2% (32/349), respectively (**Table 1**).

In univariate analysis, PLNs, SCCA levels of ≥2.55 ng/mL, tumor size of ≥4 cm, DSI, positive LVSI, parametrial invasion, and no ART were predictors of LR and DM (**Table 2**). In multivariate analysis, DSI (odds ratio [OR] = 1.494; 95% confidence interval [CI]: 1.286–2.853, *P* = 0.011) and ART (OR = 0.148; 95% CI: 0.075–0.291, *P* = 0.000) were independently associated with LR. LVSI (OR= 1.928; 95% CI: 1.151–3.229, *P* = 0.013) and adjuvant chemotherapy (OR = 0.521; 95% CI: 0.317–0.733, *P* = 0.040) were independently associated with DM. In addition, PLNs (OR = 3.729; 95% CI: 1.838–7.563, *P* = 0.000) and ART (OR = 0.470; 95% CI: 0.176–0.843, *P* = 0.003) were independently associated with LNR. SCCA levels of ≥2.55 ng/mL (OR = 1.642; 95% CI: 1.325–2.265, *P* = 0.002) and LVSI (OR = 1.462; 95% CI: 1.203–2.048, *P* = 0.005) were independently associated with organ recurrence (**Table 3**).

**Table 3:** Multivariate logistic regression models for different recurrence sites and subtypes.

Subgroup	Reference	LR		DM		LNR		Organ recurrence	
		OR (95CI)	P	OR (95CI)	P	OR (95CI)	P	OR (95CI)	P
**Tumor size**									
>=4cm	<4cm	1.308 (0.808~2.118)	0.274			1.012 (0.558~1.835)	0.970	1.016 (0.563~1.833)	0.958
**Lymph nodes**									
N+	N−	1.159 (0.670~2.005)	0.598	1.622 (0.962~2.735)	0.070	3.729 (1.838~7.563)	<0.001	1.559 (0.791~3.072)	0.200
**SCCA**									
>=2.55	<2.55	1.265 (0.739~2.164)	0.392	1.127 (0.678~1.872)	0.645	1.509 (0.762~2.991)	0.238	3.690 (1.838~7.407)	<0.001
**DSI**									
>=1/2	<1/2	2.026 (1.172~3.502)	0.011	1.792 (0.908~3.534)	0.093	1.698 (0.509~5.682)	0.388	1.743 (0.532~5.709)	0.358
**LVSI**									
Positive	Negative	1.747 (0.763~4.001)	0.187	1.931 (1.153~3.235)	0.012	2.140 (0.936~4.893)	0.071	2.367 (1.154~4.919)	0.002
**Parametrial**									
Positive	Negative	1.293 (0.671~2.490)	0.443	1.212 (0.618~2.377)	0.576	1.191 (0.591~2.397)	0.625	1.143 (0.572~2.287)	0.705
**ART**									
Yes	No	0.148 (0.075~0.291)	<0.001			2.401 (1.470~4.543)	<0.001	0.650 (0.212~1.993)	0.451
**Chemotherapy**									
Yes	No			0.519 (0.31~0.869)	0.043	1.078 (0.373~3.114)	0.890	0.895 (0.312~2.567)	0.837

SCCA, squamous cell carcinoma antigen; LVSI, lymph-vascular space invasion; DSI, deep stromal invasion; ART, adjuvant radiotherapy.

### Survival and Stratification Analyses

**Figure 1:**
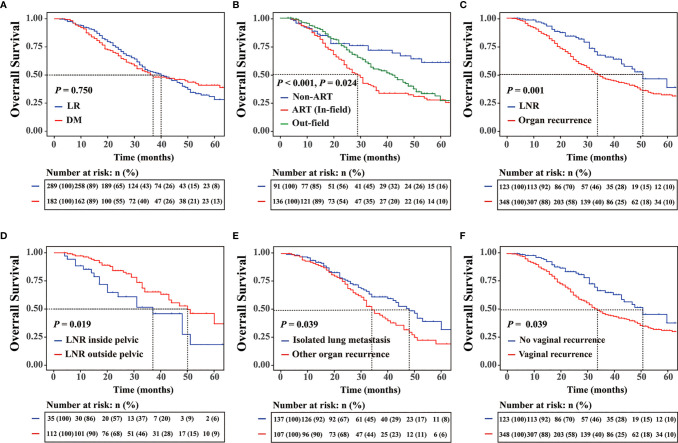
**(A)** Five-year overall survival (OS) rates for different recurrence sites. **(B)** OS rates for patients with locoregional recurrence (LR) with or without adjuvant radiotherapy (ART) and the OS for patients with the out-field recurrence. **(C)** Five-year OS rates for patients with different recurrence subtypes, **(D)** patients with lymph node recurrence, **(E)** patients with or without isolated lung metastasis, and **(F)** patients with or without vaginal stump.

For cases of disease recurrence, the median follow-up time was 59.1 (range 1.9–146.6) months. The 5-year OS rate was 33.7%, and the median OS was 24.0 months. Patients with LR (5-year OS rate, 28.1%; median OS = 40 months) and those with DM (5-year OS rate, 38.9%; median OS = 37 months) (*P* = 0.755) had similar survival rates (**Figure 1A**). However, the prognosis was poorer for patients with LR with ART than for those without ART (**Figure 1B**). LNR (5-year OS rate, 38.3%; median OS = 51 months) had a better survival than organ recurrence (5-year OS rate, 30.7%; median OS = 34 months) (*P* = 0.001) (**Figure 1C**). Prognosis associated with pelvic LNR (5-year OS rate, 18.3%; median OS = 37 months) was poorer than that associated with LNR outside of the pelvis (5-OS rate, 36.9%; median OS = 50 months) (*P* = 0.019) (**Figure 1D**). Among patients with organ recurrence only, lung metastasis (5-year OS rate, 32.5%; median OS = 48 months) was associated with survival rates that were better than those associated with other organ recurrence sites (5-year OS rate, 19.7%; median OS = 34 months) (*P* = 0.039) (**Figure 1E**). Patients with vaginal recurrence (5-year OS rate, 31.7%; median OS = 34 months) had a worse prognosis than did those without vaginal recurrence (5-year OS rate, 46.0%; median OS = 51 months) (*P* = 0.001) (**Figure 1F**). According to ART stratification, in-field recurrence was associated with poorer prognosis (5-year OS rate, 24.9%; median OS = 29 months) than out-field recurrence (5-year OS rate, 30.8%; median OS = 42 months) (*P* = 0.024) (**Figure 1B**). For all recurrent cases, surgery was associated with 5-year OS rates that were better than those associated with RT and chemotherapy (5-year OS rate of surgery, RT and chemotherapy: 56.9%, 35.7% and 27.3%; median OS: NA, 46 months and 32 months, respectively, P < 0.001). For in-field recurrence only, surgery (5-year OS rate, 36.5%; median OS = 36 months) was associated with OS that was better than that associated with secondary RT (5-year OS rate, 12.2%; median OS = 24 months) (*P* = 0.047). In patients with LR, LNR, and organ recurrence, outcomes associated with surgery and RT were better than those associated with systemic chemotherapy (*P* = 0.011).

### Predictive Factors for OS and Nomogram

**Figure 2:**
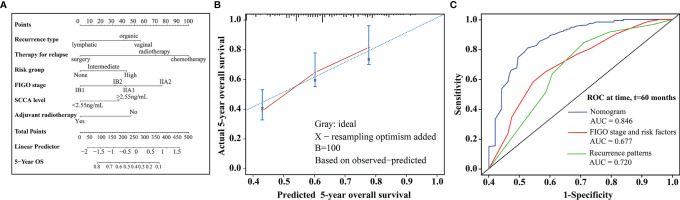
**(A)** Nomogram predicting 5-year OS rates for patients with relapse after initial treatment. The nomogram was based on scores corresponding to each independent variable. The total score (bottom of the scale) indicates the probabilities of 5-year OS rates. **(B)** The predicted and observed 5-year OS rates were used for model calibration. The x-axis displays nomogram-predicted probability, while the y-axis displays observed survival rates estimated using the Kaplan-Meier method. The dotted line indicates excellent model calibration, with good concordance between the predicted and observed survival rates. The vertical bars represent 95% confidence intervals. **(C)** Receiver operating characteristic curves with area under the curve values compare nomogram and traditional model discrimination. The blue lines represent survival rates predicted by the nomogram.

In multivariate analysis, type of recurrence, therapy for relapse, the FIGO stage, ART, baseline serum SCCA levels, and risk factor subgroup were independently associated with prognosis after recurrence (**Table 4**). All relevant predictors were used to construct a prognostic nomogram, and points were assigned based on corresponding factor coefficients; the total score was used to predict 5-year OS rates. The C-index of the nomogram was 0.724 (95% CI, 0.679–0.769) in the internal validation set (**Figure 2A**). Calibration curves are presented in **Figure 2B**. The results indicated that the nomogram was well-calibrated. In addition, this nomogram was compared to commonly used risk prediction methods, including the FIGO stage and Sedlis criteria and other previously used models. The AUC of our nomogram was greater than those of models previously used (0.846 vs. 0.720 vs. 0.677, *P* < 0.001) (**Figure 2C**).

**W 4:** Multivariate Cox proportional hazards models for 5-year overall survival.

Subgroup	Reference	HR	95 CI	*P* Value
**Recurrence type**				
Organ recurrence	Lymph nodes recurrence	1.917	1.151-3.193	0.012
Vaginal recurrence		2.448	1.661-3.609	<0.001
**Therapy for relapse**				
Chemotherapy	Radiotherapy	1.105	0.786-1.553	0.567
Surgery		0.500	0.275-0.907	0.023
**FIGO stage**				
IB2	IB1	2.193	1.292-3.721	0.004
IIA1		1.526	0.995-2.342	0.053
IIA2		2.043	1.331-3.138	0.001
**ART**				
Yes	No	0.567	0.404-0.795	0.001
**Risk group**				
Intermediate	None	2.073	1.653-4.036	0.002
High		2.155	1.649-4.470	0.003
**SCCA**				
>=2.55	<2.55	1.568	1.098-2.238	0.013

SCCA, squamous cell carcinoma antigen; ART, adjuvant radiotherapy.

## Discussion

In the present study, primary recurrence patterns were associated with the prognosis of squamous cervical cancer after initial treatment. The patterns of recurrence in cervical cancer vary, and thus, different treatments for relapse may result in different prognoses. The National Comprehensive Cancer Network Guidelines recommend individualized therapy for relapse, including surgery, RT, and chemotherapy. For localized recurrence after initial treatment, radical retreatment, including RT and/or chemotherapy, and surgery may be administered. However, radical retreatment options may vary among recurrence sites; for example, patients with regional LN recurrence may be suitable candidates for radical surgery and RT. In such cases, patients eligible for radical treatment may achieve long-term disease-free survival rates of approximately 40% (14). Most previous studies evaluated prognosis based on patients’ clinicopathological features and recurrence sites. However, few studies have examined the relationship between specific parameters and recurrence patterns. The present study examined the prognostic relevance of recurrence patterns and subtypes, and that of clinicopathological factors.

In our cohort, there were more cases of DM than LR (61.2% vs. 38.8%). Furthermore, the incidence of organ recurrence was higher than that of LNR. Vaginal recurrence was the most common recurrence site, followed by the lung and bone. Previous population-based studies reported vaginal recurrence as the most common local recurrence site in patients with cervical cancer; meanwhile, the lung and bone were the common sites of distant metastases (15, 16). In our study, LNR was a particular kind of recurrence pattern; in addition, approximately 12.5% and 8.3% of recurrence cases were observed at para-aortic and supraclavicular LNs, respectively. The para-aortic lymphatic system is connected to the cervix and pelvic LNs, resulting in a high recurrence rate in para-aortic LNs (17, 18). Kim et al. reported rates of 59.5% and 40.5% for distant and pelvic recurrence, respectively (combined: 21.5%, central: 10.7%, pelvic 8.3%) (19). Pamela et al. used PET scans to detect recurrences in para-aortic LNs and reported a rate of 18.7%, which was consistent with the present study findings (20). Tae et al. reported a 5.4% recurrence rate in the supraclavicular region after radical hysterectomy in patients with early-stage cervical cancer (21).

Common prognostication methods include the FIGO staging system and the Peters and Sedlis criteria. However, these models fail to account for the effects of recurrence patterns. When patients experience disease recurrence, little attention is given to baseline clinical characteristics, which may help assess the risk of recurrence. Studies on the relevance of recurrence patterns and initial treatment types to the risk of relapse are rare. Nevertheless, baseline clinicopathological characteristics may help inform treatment as these factors may affect the risk of relapse. Consequently, we examined the associations among baseline clinicopathological characteristics, recurrence patterns, therapy for relapse, and prognosis to establish a predictive model to inform clinical practice.

In the present study, approximately half of the patients with recurrence had elevated serum SCCA levels at diagnosis and half had positive LN status after surgery. All cases with recurrence presented with intermediate- or high-risk factors or both at baseline. Different recurrence patterns may be associated with specific clinicopathological characteristics at initial treatment. According to univariate and multivariate analyses, DSI and the absence of ART were associated with a high risk of LR. Patients who did not receive adjuvant chemotherapy but had LVSI were more likely to experience DM than their counterparts. PLN status after initial treatment or lack of ART may result in LNR. Preoperative serum SCCA levels and positive LVSI findings were independent predictive factors for organ recurrence. Jeong et al. reported that approximately 59% of recurrence cases had elevated serum SCCA levels at diagnosis (22). Serum SCCA levels are biomarkers commonly used for auxiliary diagnosis and surveillance in cervical cancer; elevated serum SCCA levels are associated with the extent of the disease (23, 24). However, no previous study has examined the association between serum SCCA levels and specific recurrence patterns. According to the Sedlis criteria, DSI is an intermediate risk factor for recurrence in cervical cancer; we have previously shown that DSI may be independently associated with both DFS and OS (25). The effect of LVSI on early-stage cervical cancer remains controversial, and previous studies have yielded conflicting results. Balaya and Obrzut found that LVSI may be associated with decreased 5-year DFS and 10-year OS (26, 27). Meanwhile, Creasman et al. have suggested that LVSI may not be a prognostic factor (28). In the present study, patients with positive LVSI had increased risk of DM, specifically, at distant organs. The present study model was superior to previous models for disease prognostication (29–31).

The prognosis associated with LR was comparable to that associated with DM. More specifically, cases of LNR had better prognosis than cases of organ recurrence; however, pelvic LNR had poor overall prognosis. Isolated LNR may be radically treated with either surgery or RT; in contrast, organ recurrence is difficult to treat with any radical approach. For patients with organ recurrence, vaginal recurrence was associated with poor prognosis; meanwhile, isolated lung metastasis was associated with better OS rates compared to those associated with the other sites of organ recurrence. Isolated lung disease may be treatable with surgery or RT, which may improve prognosis. Previous studies have shown that patients who benefit from aggressive local therapy for oligometastatic disease include those with nodal or lung metastases (32). Nevertheless, pelvic recurrence was associated with poor prognosis in patients with LR after ART. The palliation of pelvic recurrences is difficult at previously irradiated sites that are not amenable to local pain control techniques or surgical resection. These sites are generally not responsive to chemotherapy; consequently, affected patients are often advised to undergo pelvic exenteration or receive systematic chemotherapy; however, pelvic exenteration is a complex procedure susceptible to complications, which may affect prognosis. Thus, the National Comprehensive Cancer Network guidelines recommend pelvic exenteration for very select patients. Moreover, secondary RT is not feasible due to the high risk of adverse effects and dose-limiting toxicity (33, 34). Chemotherapy is often recommended for patients with extra-pelvic metastases or recurrent disease who are not candidates for RT or exenterative surgery.

This study had some limitations that should be considered when interpreting its findings. First, it was a retrospective study, which makes it subject to the effects of selection bias and confounding factors. Second, nodal metastasis has been redefined in the 2018 FIGO staging system; however, this study dataset and the included citations refer mostly to the 2009 FIGO staging system. Finally, although local treatment status emerged as a prognostic factor for OS, the lack of precise information on recurrence treatment may impact the specificity of the present results.

## Conclusion

Recurrent cervical cancer is associated with poor prognosis in cases of in-field recurrence. Different clinicopathological characteristics are associated with different recurrence sites and subtypes. The present findings suggest that patients with DSI and absence of ART are at a high risk of LR. Patients who did not receive adjuvant chemotherapy and presented with LVSI were more likely to experience DM than their counterparts. PLNs after initial treatment or lack of ART may increase the risk of LNR. Preoperative serum SCCA levels and positive LVSI status increased the risk of organ recurrence. Recurrent cervical cancer prognosis is associated with recurrence location and subtype.

## Data Availability Statement

The raw data supporting the conclusions of this article will be made available by the authors, without undue reservation.

## Ethics Statement

Written informed consent was obtained from the individual(s) for the publication of any potentially identifiable images or data included in this article.

## Author Contributions

Conception and design: ZZ, LJ, and JZ. Collection and assembly of data: ZZ and LJ. Data analysis and interpretation: ZZ, LJ, and JZ. Pathological slides reviewing: RB. Manuscript writing: ZZ and LJ. Final approval of manuscript: ZZ, LJ, RB, XW, GK, and JZ. All authors contributed to the article and approved the submitted version.

## Funding

The present study was supported by the National Natural Science Foundation of China (Youth Fund no. 81902640).

## Conflict of Interest

The authors declare that the research was conducted in the absence of any commercial or financial relationships that could be construed as a potential conflict of interest.

## Publisher’s Note

All claims expressed in this article are solely those of the authors and do not necessarily represent those of their affiliated organizations, or those of the publisher, the editors and the reviewers. Any product that may be evaluated in this article, or claim that may be made by its manufacturer, is not guaranteed or endorsed by the publisher.
